# Evaluation of computational tools for the prediction of CRISPR/SpCas9 gRNA activity in plants

**DOI:** 10.1007/s00299-026-03820-x

**Published:** 2026-04-24

**Authors:** Zheng Gong, Mengyi Chen, Hui Zhang, Jenny C. Mortimer, José R. Botella

**Affiliations:** 1 School of Agriculture, Food and Wine, Waite Research Institute, Adelaide University, Glen Osmond, Adelaide, SA 5064 Australia; 2 ARC Centre of Excellence in Plants for Space, Adelaide University, Glen Osmond, Adelaide, SA 5064 Australia; 3 Plant Genetic Engineering Laboratory, School of Agriculture and Food Sustainability, The University of Queensland, Brisbane, QLD 4072 Australia; 4 Shanghai Collaborative Innovation Center of Plant Germplasm Resources Development, College of Life Sciences, Shanghai Normal University, Shanghai, 200234 China

**Keywords:** CRISPR, Genome editing, gRNA design, Computational predictions, *Nicotiana benthamiana*

## Abstract

CRISPR/Cas9 technologies are now routinely used in plant research, with guide RNA (gRNA) design being a critical determinant of genome editing success. However, rational design of highly active gRNAs is challenging due to complex sequence and biochemical factors affecting activity. While numerous computational prediction tools have been developed, they are predominantly trained on animal cell or microbial data and their performance in plants remains controversial or untested. In this study, using two independent *Nicotiana benthamiana* experimental datasets comprising a total of 52 gRNAs, we systematically evaluated over 20 freely accessible, Web-based *in silico* tools for predicting gRNA on-target efficiency. We identified several machine learning-based tools that showed strong correlation with experimental editing efficiency across both datasets. Importantly, gRNAs in the top quartile by prediction score produced significantly higher InDel frequencies than those in the lowest quartile for all tools tested. Furthermore, several algorithms available through *CRISPOR*, a platform containing a large number of non-model plant genomes, also showed good predictive performance. This may enable better integration of on-target and off-target predictions in gRNA design. Our findings provide practical guidance for improving gRNA design in plant genome editing applications.

**Supplementary Information:**

The online version contains supplementary material available at 10.1007/s00299-026-03820-x.

In class II CRISPR systems, such as CRISPR/SpCas9, the guide RNA (gRNA) is not only a critical contributor to target specificity (Jinek et al. 2012; Liang et al. 2016; Mao et al. 2019) but is also a crucial, underlying factor affecting genome editing (GE) efficiency (Moreb and Lynch 2021). GE efficiency can be very variable for different gRNA targets, and designing active gRNAs is paramount for a successful GE workflow, especially for plants with laborious and time-consuming transformation procedures (Gong et al. 2025; Slaman et al. 2023; Naim et al. 2020; Mao et al. 2019; Moreb and Lynch 2021). Manual design of effective gRNAs is challenging due to the complicated sequence and biochemical factors that govern on-target activity (Chen and Wang 2022; Konstantakos et al. 2022; Liang et al. 2016; Moreb and Lynch 2021). Multiple computational-based tools are available for the prediction of gRNA activity, many of which were developed using machine learning (ML), but were largely not developed for plant systems (Konstantakos et al. 2022; Yan et al. 2018). Thus, the applicability of gRNA efficiency prediction in plant genome editing is still controversial (Liang et al. 2019; Naim et al. 2020; Slaman et al. 2023). Here, we systematically evaluated the performance of CRISPR/SpCas9 gRNA on-target efficiency prediction tools (listed in Table S1 with references) and identified several ML-based tools that were effective in predicting the experimental activity of gRNAs in the model plant, *Nicotiana benthamiana*.

We chose to generate experimental GE datasets through transient expression of SpCas9 and gRNAs in *N. benthamiana* leaves (Fig. 1A) over stable transformation approaches due to its inherently higher throughput and, quantitative nature, which captures subtle variations in GE efficiency between gRNAs and provides an assessment of the influence of gRNA spacer sequences. Using leaf agroinfiltration with individual gRNAs, we generated two independent experimental datasets in two different laboratories. Briefly, the first dataset was described in our recently published study (Gong et al. 2025) where we designed 20 CRISPR/SpCas9 gRNA spacers targeting six different genes. The second dataset included 32 additional gRNAs targeting 19 different genes. In both cases, GE efficiency was quantified as the frequency of reads with InDel mutations determined by targeted amplicon sequencing (AmpSeq) (Figure S1 for dataset 1 and Fig. 1B for dataset 2). We initially used these datasets to infer potential underlying factors influencing gRNA activity in plants as described in Supplementary Notes 1. Nevertheless, gRNAs in both datasets generated a similar spectrum of InDel frequencies (Fig. 1C), but the latter was more skewed, possibly because its gRNAs were manually designed without input from prediction tools unlike dataset 1. Furthermore, despite transient expression-based assays being generally reproducible as observed when testing the same gRNA in independent experiments (Figure S2), it is important to note that our datasets are notably smaller than those used in mammalian studies (Chen and Wang 2022; Labuhn et al. 2018) and are more vulnerable to variability and noise.

**Fig. 1:**
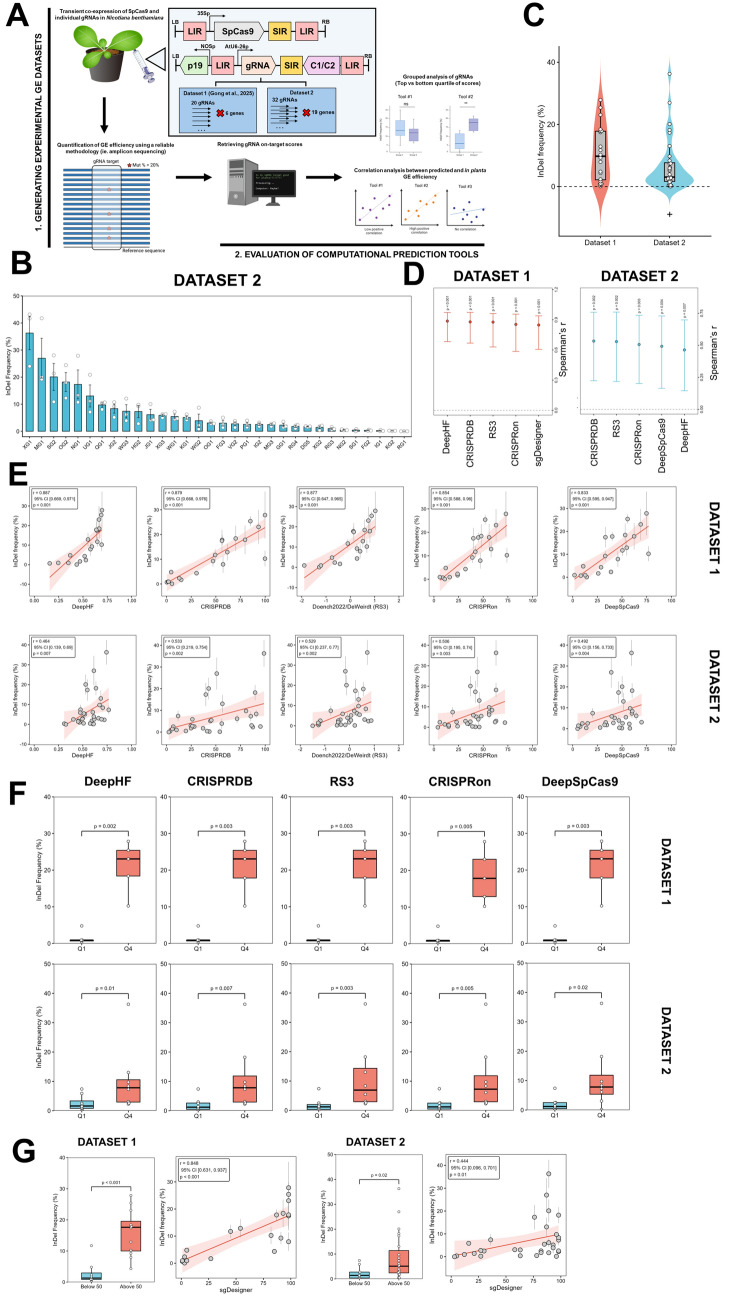
Systematic evaluation of developed gRNA on-target prediction tools. **A** Schematic representation of the workflow for this study (Created with BioRender.com; Credit to DBCLS for tobacco plant: Bioicons.com). The two experimental genome editing datasets were generated through transient coexpression of SpCas9 and individual gRNAs using geminiviral-based replicons in *Nicotiana benthamiana* leaves as described in Gong et al. (2025). Dataset 1 was previously generated by Gong et al. (2025) and Dataset 2 was generated in this study. Genome editing efficiency was quantified using targeted amplicon sequencing (AmpSeq). Efficiency prediction scores for these gRNAs were then retrieved, and linear regression and correlation analyses were conducted to determine the relationship between gRNA on-target prediction scores and their *in planta* activity. The gRNAs were then categorized into groups (i.e., quartiles) based on their prediction scores and the overall genome editing efficiencies between the two groups were compared. **B** Bar graph showing the frequency of reads with CRISPR-mediated InDel mutations across 32 gRNAs in dataset 2 as quantified using AmpSeq. Bars represent the mean InDel frequency across biological replicates ± SEM. Data points represent the quantified frequency of reads with InDels of each biological replicate. **C** Box and whisker plot overlaid with a violin plot showing the distribution of mean InDel frequencies across the 20 gRNAs in dataset 1 (red) and 32 gRNAs in dataset 2 (blue). The solid line represents the median. Each data point represents the mean InDel frequency across biological replicates of a gRNA. + , The InDel frequency of gRNAs in dataset 2 showed a skewed distribution according to the D’Agostino test of skewness (p-value < 0.001) while dataset 1 did not (*p*-value = 0.3691). **D** Forest plot of the top five efficiency prediction tools showing the highest Spearman’s *r* for gRNAs in dataset 1 (left) and dataset 2 (right). Note that *AIdit-CRISPR*, a top-performing tool was excluded from this plot because it was no longer accessible (December 2025). The whiskers represent bootstrap 95% confidence interval (CI) around the Spearman’s *r* with statistical significance shown as a *p*-value for each tool. **E** Linear regression and correlation analyses of scores from five top-performing efficiency prediction tools and GE efficiency in dataset 1 (top) and 2 (bottom). Each data point represents the mean InDel frequency ± SEM of a gRNA and its corresponding prediction score. The regression line is shown in red and the shaded region represents 95% CI for linear regression. The Spearman’s* r*, bootstrap 95% CI of the correlation and p-value were calculated and shown. **F** Box and whisker plots representing the CRISPR-mediated InDel frequencies of gRNAs categorized into the lowest quartiles (Q1) and the top quartile (Q4) based on the assigned scores for the five top-performing tools for dataset 1 (top) and dataset 2 (bottom). An unpaired *t*-test was conducted to determine whether differences in InDel frequencies between the two groups were statistically significant, while a Mann–Whitney *U* test was used for dataset 2. **G** Evaluating the *sgDesigner* tool for predicting gRNA on-target efficiency across the two datasets (dataset 1 and 2 in the left and right panels). Box and whisker (left of each panel) of the grouped analysis of *sgDesigner* where gRNAs were categorized into groups with scores above or below 50. An unpaired *t*-test was used to determine whether differences in InDel frequencies between the two groups were statistically significant for dataset 1, and a Mann–Whitney *U* test was used for dataset 2. Linear regression and correlation analysis (right of each panel) of *sgDesigner* score and genome editing efficiency in plants across the gRNAs in the two datasets. Each data point represents the mean InDel frequency ± SEM of a gRNA and its corresponding prediction score. The regression line is shown in red and the shaded region represents 95% CI for linear regression. The Spearman’s* r*, bootstrap 95% CI of the correlation, and *p*-value were calculated and shown

We initially queried the *CRISPOR* Web-based platform to evaluate the popular *Doench2016 (Rule Set 2 or RS2)* and *Moreno-Mateos* efficiency prediction scores with dataset 1 (Figure S6). We found that *Doench2016* scores showed a positive and statistically significant correlation with genome editing efficiency (Figure S6A). The groups of gRNAs with *Doench2016* scores in the top quartile produced significantly higher InDel frequencies compared to the group of gRNAs in the lowest quartile. By comparison, the *Moreno-Mateos* score was not effective for the same task (Figure S6B). Unfortunately, we did not find a statistically significant correlation between the *Doench2016* score and the GE efficiency of gRNAs in our second dataset (Figure S6C). One potential cause of this variability in performance across the two datasets may be due to the increased skewness with more inefficient gRNAs observed in dataset 2. The 32 designed gRNAs within this dataset targeted nearly 20 different genes in which epigenomic variation, known to affect on-target efficiency (Weiss et al. 2022), was not accounted for. We explore this further in Supplementary Notes 2 and 3. Nonetheless, the unsatisfactory performance of these two efficiency scoring algorithms across our two datasets prompted us to further explore *other in silico* gRNA efficiency prediction tools.

Next, using our first dataset, we evaluated 19 gRNA efficiency prediction tools (detailed in Table S1) of which most were untested in plants. The evaluation of these tools in plants is crucial as most of the tested ML-based tools were trained on datasets of gRNA sequence and performance in animal systems, while others like *E-CRISP* and *CRISPR-P v2.0* relied on hypothesis-driven and rule-based scoring. Notably, the selection of reference genomes used to run these tools, as described in the Supplementary Methods, would mostly influence off-target analysis rather than the prediction of on-target efficiency. Intriguingly, many of the prediction scores from the evaluated tools showed a statistically significant correlation with experimental, *in planta* GE efficiency (Fig. 1D). In particular, we identified six ML-based tools which showed high correlation (Spearman’s* r* over 0.8) (Figs. 1E and S7 for *AIdit-CRISPR*). We performed a Steiger’s *Z*-test for pairwise comparison of tools to determine whether differences between correlations have statistical significance (Supplementary Data 4). Overall, we found that these six top-performing tools did not differ significantly, but several of these tools outperformed other efficiency scoring systems. For example, we saw a significantly higher Spearman’s *r* for *CRISPRDB*, *Doench2022/DeWeirdt* (*Rule Set 3* or *RS3*), *CRISPRon* and *DeepSpCas9* when compared to tools like *CRISPRedict* and *CCTop*. In addition, we observed that the scores from these three tools also predicted the spectrum of activity across gRNAs in the dataset. Meanwhile, despite showing a high correlation, a portion of gRNAs were assigned very similar prediction scores by *DeepHF* and data points clustered together rather than dispersed.

Encouraged by these results, we interrogated five of these six well-performing tools with our second dataset (*AIdit-CRISPR* was not accessible, December 2025). The gRNA efficiency prediction scores of all 5 assessed tools showed a statistically significant correlation with *in planta* GE efficiency (Fig. 1E). *CRISPRDB* showed the highest Spearman’s* r* of around 0.53 with other tools showing correlations of around 0.5, but this difference was not statistically significant based on the Steiger’s *Z*-test (Supplementary Data 6). Despite showing robust correlation with experimental InDel frequencies, the Spearman’s* r* for these tools was, overall, lower than that in dataset 1, and showed wide bootstrap 95% confidence intervals (Fig. 1D and E). Such variability in tool performance between datasets is generally expected but has likely been greatly amplified due to our smaller datasets with a limited number of gRNAs and, in particular, significant skewness from our second dataset. To further confirm the ability of the efficiency predictions in differentiating effective and ineffective gRNAs in plants, we split gRNAs into quartiles and found that ones with scores in the top quartile did, indeed, produce significantly higher experimental InDel frequencies compared to the lowest quartile for all five tools in both datasets (Fig. 1F).

Another interesting tool was *sgDesigner* with its prediction scores showing a statistically significant correlation with on-target GE efficiency in both datasets, but it assigned either very high or low scores to gRNAs and lacked variance (Fig. 1G). By categorizing gRNAs into groups, we found that gRNAs with *sgDesigner* scores above 50 mediated a significantly higher frequency of InDels compared with those below 50 (Fig. 1G). All 12 gRNAs with *sgDesigner* scores above 50 mediated detectable levels of InDels in dataset 1. Thus, *sgDesigner* scores may be useful for selecting functional gRNAs but further investigation with a larger number of gRNAs is needed for confirmation.

Apart from efficiency, specificity is also critical in GE experiments where many online tools have included genome information to identify potential off-target sites. *CRISPOR* and *CRISPR-P v2.0* are two useful gRNA design portals containing many non-model plant genomes, which are often missing or limited in other prediction tools. Besides tested ones like *Doench2016*, *Moreno-Mateos* and *RS3*, we used dataset 1 to evaluate the six remaining efficiency scoring systems on *CRISPOR*. The *Chari*, *Azimuth* in-vitro (no longer accessible) and *Wang* prediction scores showed the highest correlation with *in planta* GE efficiency in dataset 1 (Spearman’s* r* = 0.76, 0.74, and 0.69, respectively) and could serve as useful tools for gRNA design, especially for non-model plants (Figure S9). For dataset 2, *Chari* and *Wang* scores were also moderately correlated (*r* = 0.388 and 0.398, respectively) with gRNA activity (Figure S10). Despite being less correlative, this difference was not statistically significant compared to *CRISPRDB*, likely due to the large bootstrap 95% CI across all Spearman’s *r* in this dataset. Simultaneously, we showed that groups of gRNAs with higher *Chari* score did not mediate more effective genome editing in dataset 2 as compared to dataset 1 while the *Wang* score was capable of distinguishing efficient and inefficient gRNAs in both datasets (Figures S9 and S10). On the other hand, the *CRISPR-P v2.0* prediction scores were much less correlative with experimental efficiency in dataset 1 when compared to other tested tools such as *CRISPRDB*, *RS3*, *CRISPRon* and several ones on *CRISPOR* (Supplementary Data 4). We did not continue to test other tools on *CRISPOR* as well as *CRISPR-P v2.0* with dataset 2. Analysis of remaining tools that were evaluated using dataset 1 is shown in Figure S11 with Steiger’s *Z*-tests for pairwise comparison of the Spearman’s *r* between tools (Supplementary Data 4).

We attempted to further improve efficiency prediction by combining the outputs from prediction tools evaluated with dataset 2. The scores were quantile normalized and averaged to obtain a combined or ensemble score. In this way, we produced efficiency scores for gRNAs by combining outputs from two to four prediction tools (Figure S12). Overall, we saw minor increases in correlation for several combinations of the five top-performing tools, but the differences had no statistical significance as shown in a Steiger’s *Z*-test that compared them to *CRISPRDB* alone (described further in Supplementary Notes 4). Despite this, such an approach in integrating the predictions of several tools may be a strategy to improve reliability for effective gRNA design in plant applications.

In summary, we evaluated the performance of a broad suite of gRNA efficiency prediction tools in plants with two experimental GE datasets containing a total of 52 gRNAs. As a proof-of-concept, our study showed that multiple highly accessible computational tools produced efficiency prediction scores that correlated with gRNA activity *in planta*. It is, nevertheless, important to consider that our experimental dataset consisted of a limited number of gRNAs (20 and 32 gRNAs across the two datasets). This reduces confidence in our observations, and datasets with larger numbers of gRNAs are necessary to make robust evaluations. Our dataset was also derived from a single plant species and, hence, the performance of prediction tools needs to be further validated in other plant systems. Future contributions from researchers with established transient expression-based CRISPR genome editing workflows in other plant species are needed for cross-species validation of our findings and to identify other effective gRNA on-target prediction tools to benefit future genome editing pursuits in plants. Advancements in high-throughput CRISPR screens in plants may also allow further exploration using larger sets of gRNAs and retraining of well-performing ML-based efficiency prediction algorithms to develop plant-tailored tools.

## Supplementary Information

Below is the link to the electronic supplementary material.

Supplementary file1 (XLSX 50 KB)

Supplementary file2 (DOCX 1689 KB)

## Data Availability

All experimental data generated by Gong et al. (2025) for dataset 1 and the pooled and demultiplexed AmpSeq reads from dataset 2 have been deposited in FigShare (10.25909/30932480). All information relating to the gRNA targets, analyzed genome editing data, efficiency prediction scores and Steiger’s *Z*-test statistics are provided in the Supplementary Data. The vector sequences used in this study have been previously reported by Gong et al. (2025). Primer sequences for amplicon sequencing of gRNA targets in dataset 2 have been deposited into the same FigShare folder, alongside metadata required for batch analysis. Any other data or information required for reanalysis will be made available upon request.
