# Supplementary material for: Evaluation of computational tools for the prediction of CRISPR/SpCas9 gRNA activity in plants
Source: Plant Cell Rep. 2026 Apr 24;45(5):136. doi: 10.1007/s00299-026-03820-x (PMC13109197; doi:10.1007/s00299-026-03820-x)
Supplement: Supplementary file 2 — Supplementary file2 (DOCX 1689 KB) [file 299_2026_3820_MOESM2_ESM.docx]

**SUPPLEMENTARY INFORMATION**

**Evaluation of computational tools for the prediction of CRISPR/SpCas9 gRNA activity in plants**

Zheng Gong^1,2,3^, Mengyi Chen^4^, Hui Zhang^4,*^, Jenny C. Mortimer^1,2,*^, José R. Botella^3,*^.

^1^Waite Research Institute, School of Agriculture, Food and Wine, Adelaide University, Glen Osmond, SA, Australia, 5064.

^2^ARC Centre of Excellence in Plants For Space, Adelaide University, Glen Osmond, Australia, SA, 5064.

^3^Plant Genetic Engineering Laboratory, School of Agriculture and Food Sustainability, The University of Queensland, St Lucia, QLD, Australia, 4072.

^4^Shanghai Collaborative Innovation Center of Plant Germplasm Resources Development, College of Life Sciences, Shanghai Normal University, Shanghai, China, 200234.

*Co-senior and corresponding authors:

Hui Zhang (zhanghui29@shnu.edu.cn)

Jenny C. Mortimer (jenny.mortimer@adelaide.edu.au)

José R. Botella (j.botella@uq.edu.au)

This document contains:

SUPPLEMENTARY NOTES 1 - 4

SUPPLEMENTARY METHODS

SUPPLEMENTARY FIGURES S1 – S12

SUPPLEMENTARY TABLE S1

REFERENCES

**SUPPLEMENTARY NOTES**

*Supplementary Notes 1. Analysis of gRNAs to devise underlying factors influencing genome editing efficiency in plants*

The 52 gRNA spacers across the two datasets showed widely variable genome editing efficiencies in *N. benthamiana* (Figure 1B, 1C, S1), emphasizing the importance of gRNA design and on-target efficiency prediction. Different gRNA sequence motifs and positional nucleotide preferences have been observed in animal systems (Xiang et al., 2021, Xu et al., 2015). Uncovering these features has been crucial for improving gRNA design and could potentially be used to develop better on-target efficiency prediction models (Doench et al., 2016, Konstantakos et al., 2022, Wang et al., 2014). We wondered whether efficient gRNAs in our study showed any positional nucleotide preferences. To test this, the gRNAs of the two datasets were split into quartiles based on their *in planta* genome editing efficiency. The two datasets were combined and spacers in the most and least efficient quartiles were used to produce sequence logos (Figure S3). Interestingly, we found slight preferences for thymine nucleotides across the gRNA spacers in the least efficient quartiles which may be due to repetitive thymines acting as a termination signal for U6 promoters, reducing gRNA expression. In contrast, gRNA spacers in the most efficient quartiles preferred purines (guanine or adenine) around the middle of the spacer. In particular, there seem to be an enrichment of purines in the 3^rd^ and 4^th^ position of efficient gRNA spacers.

To further test this, we grouped gRNAs based on the nucleotide composition at these two positions (ie. purine at position 3 and pyrimidine at position 4 = R_3_Y_4_) (Figure S4). For both datasets, we saw higher InDel frequencies in gRNAs with purines at both positions but this was only statistically significant when compared to R_3_Y_4_ and to Y_3_R_4_ for gRNAs in dataset 2. No statistically significant relationship was identified between having purines or pyrimidines at both positions. In addition, the GC content of PAM distal regions of the gRNA were suggested to influence experimental efficiency. We saw that having PAM distal GC of 25 – 50% and 51 - 75% showed higher overall InDel frequency compared to low GC % (0 – 25%) but this was not statistically significant. Likewise, we did not see many differences between groups of gRNAs of variable overall GC %. However, our study did not include any gRNAs were extreme GC content (ie. lower than 20% or higher than 80%).

Besides these, we also did not observe many other sequence motifs and nucleotide preferences in the spacer or PAM as described previously. This is consistent with previous reports by Liang et al., (2016) which we believe is possibly due to immensely smaller datasets in plants. Even though we attempted to slightly expand the number of gRNAs in our datasets, it still remains incomparable to those previously reported in other systems and is likely extremely susceptible to noise. Any observations or trends made should be treated as preliminary and assessed further using much more comprehensive datasets. Therefore, further research in developing higher throughput genome editing assays in plants is critical in understanding the underlying factors affecting gRNA activity and to develop a plant-specific gRNA efficiency prediction tool.

*Supplementary Notes 2. Prediction of gRNA activity using* Doench2016

Experimental evaluation of prediction models for gRNA on-target activity in plants was mostly studied as complements to other research in past studies. To our knowledge, there has been a lack of extensive investigation into this topic, even though research in this area has been heavily invested in animal systems (Chari et al., 2015, Chari et al., 2017, Chen and Wang, 2022, Concordet and Haeussler, 2018, Corsi et al., 2023, DeWeirdt et al., 2022, Doench et al., 2016, Doench et al., 2014, Haeussler et al., 2016). Yet, designing suitable, functional and optimal gRNA is absolutely crucial to efficient CRISPR genome editing in plants.

Several studies preceding this work have attempted to evaluate the applicability of gRNA on-target prediction tools for plant genome editing but results have been inconsistent. For example, Naim et al., (2020) transiently expressed 10 gRNAs in *N. benthamiana* to evaluate 8 gRNA efficiency prediction software but found that none were correlative. Three of the 8 tested programmes, *Benchling*, *CRISPOR-D*, *CRISPRko*, utilizes the *Doench2016* efficiency score. Contradictorily, later work in tomato protoplasts (Slaman et al., 2023) found that the *Doench2016* score may be a predictor of gRNA on-target efficiency. Likewise, our experimental GE dataset 1 did also find a significant and high correlation between *Doench2016* scores and gRNA activity *in planta* (Figure S6A). In contrast, our second GE dataset did not find any statistically significant correlation (Figure S6C). Notably, gRNAs in the first dataset were designed using *Doench2016* scores as a guideline to obtain a set of gRNAs mediating a broad spectrum of GE efficiency, whereas we manually picked gRNAs in the second dataset. This caused our second dataset to be skewed with a greater portion of inefficient or moderate gRNAs which we confirmed with a D’Agostino test of skewness. Overall, we saw poorer performance from prediction tools on gRNAs from dataset 2 with much larger bootstrap 95% confidence intervals (CI) for Spearman’s *r*.

*Doench2016* may be unable to accurately predict these as ineffective or that the factors leading to this collection of gRNAs being ineffective differs between plant and animal systems in which the model was trained on. Another possibility may be because we greatly expanded the number of genes that was targeted (from 6 genes in dataset 1 to 19 genes in dataset 2) and we did not take into account the epigenomic status at the target loci which has been shown to critically affect GE efficiency but is often not considered by efficiency prediction tools such as *Doench2016*. The effects of these factors of variability and underlying noise are essentially amplified by a small dataset that only incorporated a limited number of gRNAs. Hence, the controversy remains on whether the *Doench2016* efficiency score is a predictor of gRNA activity in plants and should be examined further with a larger dataset.

Interestingly, although now inaccessible, we found slightly improved performance with *Azimuth* in-vitro (for gRNAs in dataset 1, Figure S9) which was the *Doench2016* model re-trained on zebrafish data but this difference was not significant according to the Steiger’s *Z*-test. The successor of *Doench2016* (Rule Set 2), Rule Set 3 or what we refer to as *Doench2022/DeWeirdt* which integrated new features have offered a more robust performance in both dataset 1 and 2 (Figure 1E). Overall, these results suggest that further understanding and curation of feature sets as well as model re-training, potentially with plant-derived datasets that are larger, may lead to improvements of gRNA efficiency prediction.

*Supplementary Notes 3. Importance of genome editing quantification in studying gRNA on-target predictions*

Corsi et al., (2023) have argued that reporter gene knockout assays are less quantitative and are biased towards specific mutation types. Phenotypic screens to assess gRNA on-target efficiency that rely on the knockout of a functional gene are also dependent on the location in the gene structure that the gRNA targets (Wang et al., 2014). Further emphasis was placed on the use of accurate techniques for quantifying genome edits, such as AmpSeq, when generating experimental datasets for evaluating and training gRNA on-target prediction algorithms (Corsi et al., 2023, Xiang et al., 2021).

The impact of different quantification methods was considered by re-analyzing datasets generated previously (Gong et al., 2025) where we quantified genome edits at these same 20 gRNA targets (dataset 1) using 6 other methods: T7E1 analyzed with agarose gel electrophoresis (A), T7E1 analyzed with TapeStation (T), PCR-Capillary Electrophoresis (CE)/Indel Detection by Amplicon Analysis (IDAA), Inference of CRISPR Edits (ICE) analysis of Sanger traces with and without PeakTrace (PT+ or PT-, respectively), and digital droplet PCR (ddPCR). We performed linear regression analyses between the quantified genome editing frequencies and the gRNA’s *Doench2016* prediction scores (Figure S5). We plotted each technique with the AmpSeq benchmark on the same XY axis. The ddPCR dataset generated a linear regression line that is comparable to AmpSeq with a similar slope and R^2^ (Figure S5). In contrast, both ICE (PT+) and ICE (PT-) had very different R^2^ and slope in comparison to AmpSeq (Figure S5D, E). To our surprise, T7E1 (A) and T7E1 (T) had closer R^2^ values despite their much lower slope (Figure S5). Overall, we showed that using different techniques to quantify CRISPR genome editing may lead to variable results when evaluating the same gRNA on-target prediction tool. This may also explain differences in the outcome of studies and reinstate the importance of selecting a reliable method to assess genome editing efficiency in future work.

*Supplementary Notes 4. Combining efficiency scores from multiple tools slightly improves predictions*

We reasoned that cross-validating scores given by these tools may be a useful approach to improve confidence in the output. The 6 top-performing prediction scores (including *AIdit-CRISPR*) showed different degrees of correlation amongst each other for the 20 gRNAs in dataset 1 (Figure S8). *CRISPRDB* showed the least correlation with *Doench2022/DeWeirdt* and *DeepHF*. We tried to integrate and combine the outputted scores by normalizing and averaging the scores between prediction tools for a given gRNA. This was done by taking outputs from any combinations of two to four tools in dataset 2, perform quantile normalization for ones to be combined and then average the normalized scores. Next, we performed correlation analysis of these combination scores with the InDel frequency of gRNAs to compute the Spearman’s *r* which was compared to that of *CRISPRDB* through a Steiger’s *Z*-test. The correlation for the highest 20 combinations or single tools was plotted in Figure S12A. The linear regression and statistics of correlation analysis for the five combinations with the highest Spearman’s *r* are shown in Figure S12B.

Overall, combinations of *CRISPRDB* with *RS3* alone, with a combination of *RS3* and DeepSpCas9, or *RS3* and *CRISPRon* showed an increase in Spearman’s *r* of around 0.02 compared to *CRISPRDB* alone. This is consistent with our previous observations that these tools show a generally high correlation amongst their predicted scores for gRNAs in our dataset. Nonetheless, any improvements seen here is likely to be very preliminary and did not show statistical significance when benchmarked against *CRISPRDB*. Likewise, the large bootstrap 95% CIs associated with the Spearman’s *r*, a small and skewed dataset calls for further investigation with a larger, more well-designed gRNA collection to generate a robust testing dataset.

**SUPPLEMENTARY METHODS**

*Generation of experimental genome editing dataset 1*

In our previous study (Gong et al., 2025), we transiently expressed 20 individual gRNAs targeting 6 different genes in *Nicotiana benthamiana* and quantified genome editing efficiency using targeted amplicon sequencing (AmpSeq) as described. The raw reads from Illumina MiSeq 150 bp pair end sequencing were re-analyzed using the CRISPResso2 web tool (Clement et al., 2019). We used the default minimum homology to the amplicon of 60%, and used a minimum read filter of phred33 >20, minimum single bp quality >10. We strictly quantified Insertion-Deletion (InDel) mutations within a 1 bp window at the expected SpCas9 cut site (3 bp upstream of the PAM) and ignored substitution mutations. This was important because we noted the occurrence of minimal, but detectable levels (<1%) of substitutions in some negative control samples. An experimental plant genome editing dataset was produced with 20 gRNAs and AmpSeq-quantified InDel frequencies of 3 to 4 biological replicates each. Any samples with less than 1,000 reads were excluded from further analysis. This criterion was applied throughout the study.

*Testing the reproducibility of transient expression-based genome editing assays*

To assess the reproducibility of *N. benthamiana* leaf-based transient expression for genome editing, we selected the AG1gRNA from dataset 1 for independent testing. We co-agroinfiltrated SpCas9 and the AG1gRNA in geminiviral replicon-based vectors exactly as described in Gong et al., (2025) but in an independent experiment (Run 2). Genomic DNA was extracted, and genome edits (InDels only) were quantified using AmpSeq and CRISPResso2 as described previously (Clement et al., 2019). A nonparametric, unpaired *t*-test was used to compare and determine if differences between the mean InDel frequency of AG1 in this run and in dataset 1 (referred to as Run 1) were of statistical significance.

*Generation of experimental genome editing dataset 2*

In a separate laboratory, 32 additional gRNAs were manually designed by locating possible PAM sequences and 20 bp gRNA spacers that targeted all homeoalleles of the gene. gRNAs spacers were synthesized as forward and reverse oligonucleotides (IDT), annealed and cloned into BsaI (New England Biolabs) digested pBYR2eFa-U6-sgRNA. Vectors with cloned gRNAs were transformed into GV3101 competent cells and mixed with pIZZA-BYR-SpCas9 at an OD_600_ of 0.1 each. This is a dual geminiviral replicon-based system for robust transient co-expression of gRNAs and Cas proteins described by Gong et al., (2025). The mixture was used for agroinfiltration of leaves from 4 – 6 week old *N. benthamiana*. The *Cauliflower mosaic virus* 35S promoter was used to drive the human codon-optimized SpCas9 while an *Arabidopsis* U6-26 promoter was used to drive gRNA expression. At 7 days post agroinfiltration, leaf discs were collected from the infected region and DNA was extracted. A 200 – 300 bp region around the gRNA target was PCR amplified using gene-specific forward and reverse primers, each with a unique 10 bp barcode sequence. Different gRNA targets and amplicons were pooled together and cleaned up using the QIAquick PCR clean up kit (Qiagen) and sequenced using NovaSeq pair end 150 bp sequencing (GENEWIZ).

*Quantification of genome editing to produce dataset 2*

Pooled amplicon sequencing was de-multiplexed based on the 10 bp unique barcodes on pair end reads using cutadapt for 5’ anchored adapters, allowing for 1 mismatch, no InDels and a minimum overlap of 8 bp. Adapters were then trimmed with cutadapt and genome editing efficiency was quantified using locally run CRISPResso2 with the same settings as that used in dataset 1 (Clement et al., 2019). Briefly, we used the default minimum homology to the amplicon of 60%, ignored any substitution mutations and used a minimum read filter of phred33 >20, minimum single bp quality >10. InDel mutations within a window of 1 bp between the gRNA targets’ 17^th^ and 18^th^ nucleotides were quantified as genome edits. CRISPResso2Aggregate was used to aggregate the output of CRISPResso2 across gRNAs. We subtracted the background InDel frequency observed in the negative control (SpCas9 vector only) from all samples.

*Construction of sequence logo to identify potential positional nucleotide preferences in efficient gRNAs*

The gRNAs of each dataset were categorized into quartiles based on their on-target genome editing efficiency as measured using AmpSeq. Sequence logos were constructed using WebLogo3 (https://weblogo.threeplusone.com/). The spacer sequence and PAM (5’-NGGN-3’) of gRNAs in the most and least efficient quartile were pooled together from the two datasets and used as input to construct sequence logos.

*Retrieving gRNA on-target efficiency scores from* in silico *prediction tools*

The access portal/platform and website URL for all gRNA prediction tools that were evaluated in this study were provided in Table S1. For the *Doench2014*, *Doench2016*, *Chari*, *Xu*, *Wang*, *Moreno-Mateos*, *CCTop*, *Azimuth* *in-vitro* and *WU* scores, we queried the target sequence into the *CRISPOR* web server tool. We selected the SolGenomics *N. benthamiana* genome and the 20 bp, 5’-NGG-3’ PAM sequence for SpCas9. The gRNA sequence and its respective scores were identified and recorded. For the *Doench2022/DeWeirdt* score, we queried the target sequence on *CRISPick*. No plant genomes were available on *CRISPick*, so we selected the Human GRCh38 as our reference genome for “CRISPRko” analysis using the SpyoCas9 5’-NGG-3’ PAM sequence. Importantly, we used the “Hsu (2013)” tracrRNA for gRNA on-target prediction. The CRISPR-P v2.0 prediction scores were retrieved from the *CRISPR-P v2.0* web server using the *N. benthamiana* genome. The *CRISPRon* prediction scores of gRNAs were retrieved using the zebrafish genome. The *IDT* on-target scores were retrieved from Integrated DNA Technologies’ Custom Alt-R CRISPR-Cas9 guide RNA design tool using the *Homo sapiens* genome. The *E-CRISP* prediction scores were retrieved from the *E-CRISP* web server using the *Arabidopsis thaliana* genome and the “medium” application with “exclude targets with poly T motif” and “exclude targets with poly A motif” turned off as well as the “5’ Preceding Base requirement” changed to “any”. The *DeepSpCas9* and *DeepSpCas9variants* were both retrieved from the *DeepGE* web portal by directly entering the target gene sequence. For *DeepSpCas9variants*, prediction scores for both the SpCas9 “(G/g)N19” and “tRNA-N20” gRNAs were recorded. Notably, our study added a G nucleotide preceding the 20 bp gRNA spacer rather than shortening the sequence to 19 bp as the DeepSpCas9variants “(G/g)N19” setting specifies. The *DeepHF* prediction scores were retrieved from the *DeepHF* web server for SpCas9 gRNAs and with “SpCas9_U6” option selected. The *sgDesigner* and *CRISPRDB* prediction scores were retrieved from the *CRISPRDB* web server. Custom prediction with the “U6 promoter” settings were used. For *CRISPRedict* prediction scores, we used the “Interpret” mode and entered a 30 bp sequence around and including the gRNA target according to the manual. The “U6 promoter” and the “Regression” model were used. Lastly, for the *AIdit-CRISPR* prediction scores, the *Homo sapiens* GRCh38 genome was used with the “*AIdit_ON*” model and SpCas9 was selected as the enzyme. For all these gRNA efficiency predictions, the target sequences were manually entered, and prediction scores were retrieved and recorded.

*General statistical analysis*

All statistical analysis was performed in RStudio (release 2024.04.2+764) or in GraphPad Prism 9 as specified. Statistical significance is defined as when the p-value is lower than or equal to 0.05. A D'Agostino skewness test was performed in RStudio to determine whether the two datasets were skewed in distribution. Linear regression analysis between prediction scores assigned to gRNAs in dataset 1 was conducted in GraphPad Prism 9 where the R^2^ between the six top-performing tools and *Doench2016* was computed and shown.

*Correlation analysis between gRNA on-target prediction scores and* in planta *genome editing efficiency*

For each prediction tool, we performed a correlation analysis in RStudio to compute the reported Spearman correlation coefficients between the predicted gRNA efficiency score and *in planta* genome editing efficiency, measured as the frequency of InDels using AmpSeq. The correlation is considered statistically significant when the *p-*value < 0.05. A bootstrap 95% confidence interval (CI) around each Spearman’s *r* was calculated using the boot package with 5,000 replicates unless otherwise specified. The Steiger’s Z-test was computed using the cocor package in RStudio to compare all pairs of prediction tools and determine whether differences in correlation are of statistical significance. Briefly, the inputs of the Steiger’s *Z*-test were the individual correlation between prediction scores and the experimental InDel frequency for pairs of tools, the correlation between the prediction scores for these two tools and the sample size (number of gRNAs). The outcomes of Steiger’s *Z*-tests are reported in the Supplementary Data. The linear regression and its 95% CI was fitted using ggplot2 onto each XY plot.

*Grouped analyses by gRNA prediction scores*

Grouped analysis was used to evaluate whether prediction scores can be used to categorize more and less efficient gRNA targets. The gRNAs were categorized into quartiles based on the prediction score and an unpaired *t*-test was used to determine if differences in genome editing efficiencies between the 4^th^ quartile and gRNA in the 1^st^ quartile were of statistical significance in dataset 1. For dataset 2 which showed a non-normal distribution, a non-parametric Mann-Whitney U test was used instead. A p-value < 0.05 was considered statistically significant.

For the *WU* prediction algorithm, we categorized gRNAs with scores of 0 into one group and >0 into another group. An unpaired *t*-test was used to determine if the differences were statistically significant. For *sgDesigner*, we categorized gRNAs with scores <50 into one group and >50 into another group. Similarly, a nonparametric, Mann-Whitney U test was performed for dataset 2 and an unpaired *t*-test was used for dataset 1 to determine whether differences between groups were of statistical significance.

*Comparing CRISPR-mediated mutation quantification methods for producing experimental datasets used to evaluate gRNA on-target efficiency prediction tools*

To investigate the effect of using different genome editing quantification methods to evaluate the performance of prediction models, we retrieved the mutation frequencies for the same sample and gRNA in dataset 1 which were quantified using 6 different methods in our previous work (Gong et al., 2025). The CRISPR-mediated mutations in the 20 gRNAs and its samples were quantified using T7E1 separated using agarose gel electrophoresis (A), T7E1 separated using TapeStation (T), PCR-Capillary Electrophoresis (CE) or known as Indel Detection by Amplicon Analysis (IDAA), Sanger sequencing analyzed with Inference of CRISPR Edits (ICE) with and without base calling using Peaktrace (PT+ or PT-, respectively), and lastly with digital droplet PCR (ddPCR). We performed linear regression analysis between the quantified mutation frequency for each method across all gRNAs and its *Doench2016* prediction score. Linear regression analysis for each technique was performed using GraphPad Prism 9 and was plotted on the same XY axis with that of AmpSeq for comparison. The R^2^ and linear relationship were also computed.

*Comparing GC content and positional nucleotide preferences of gRNAs*

We investigated the nucleotide preferences at position 3 and 4 of the 20 bp gRNA spacer by manually curating the identity of nucleotides and categorizing gRNAs based on this. For the analysis of gRNAs in dataset 1, we used an unpaired *t*-test to determine whether differences in the overall experimental InDel frequency between groups were of statistically significance. For the analysis of gRNAs in dataset 2, a Mann-Whitney U test was conducted. Both tests were done in GraphPad Prism 9.

To test whether PAM distal or overall spacer GC % affects genome editing efficiency, we manually calculated the percentage of G and C nucleotides. For the PAM distal region, the 5 nucleotides spanning position 4 to 8 of the 20 bp gRNA spacer was taken for consideration. gRNAs were then categorized into groups depending on their overall GC % or GC % in the PAM distal region. The non-parametric Mann-Whitney U test was used to determine if the differences in overall InDel frequency between these groups were statistically significant.

*Combining prediction scores from multiple tools*

We reasoned that combining prediction scores from multiple tools to yield an ensemble score may improve prediction effectiveness. Prediction scores of gRNAs in dataset 2 were quantile normalized within each combination and then averaged to obtain a combined, ensemble score. Scores from two to four prediction tools were individually combined and the correlation with experimental InDel frequency was computed using RStudio. A Steiger’s *Z*-test was performed to compare the Spearman’s *r* of each combination with that of *CRISPRDB* alone which showed the highest Spearman’s *r* out of prediction tools evaluated in the second dataset.

**SUPPLEMENTARY FIGURES**


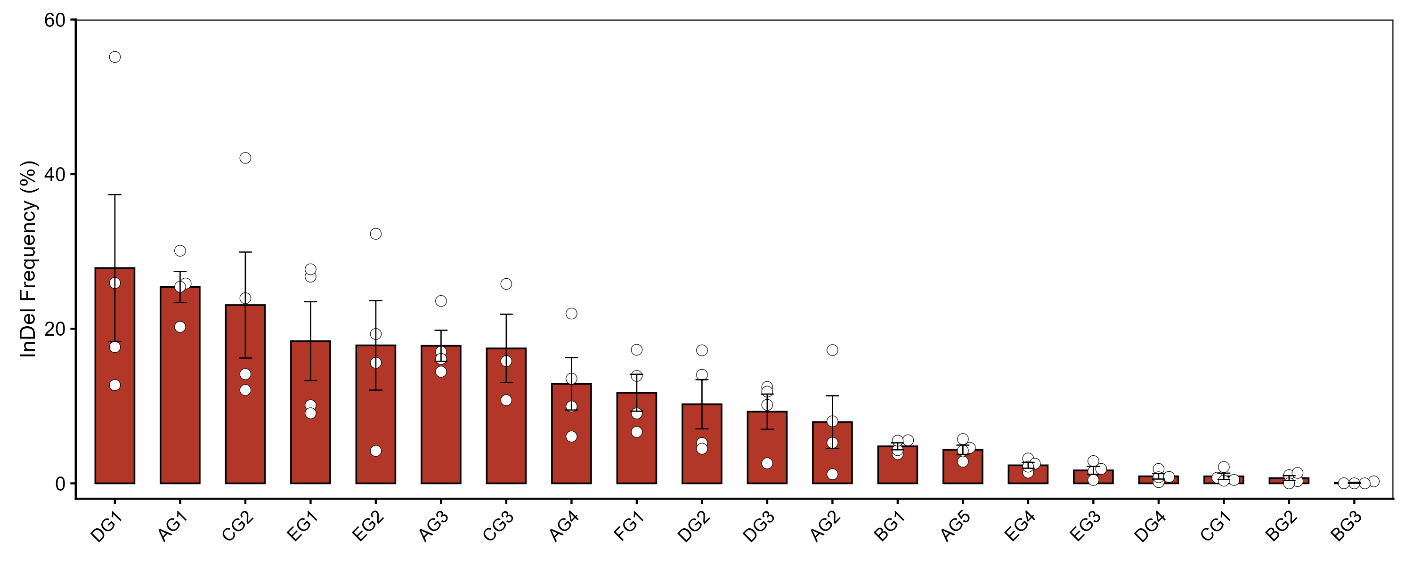


**Figure S1.** Bar graph showing the frequency of reads with CRISPR-mediated mutations across 20 gRNAs in Dataset 1 (Gong et al., 2025) as quantified using AmpSeq and was re-analyzed to capture InDels only. Bars represent the mean InDel frequency across biological replicates ± standard error of the mean (SEM). Each data point represents a single biological replicate.


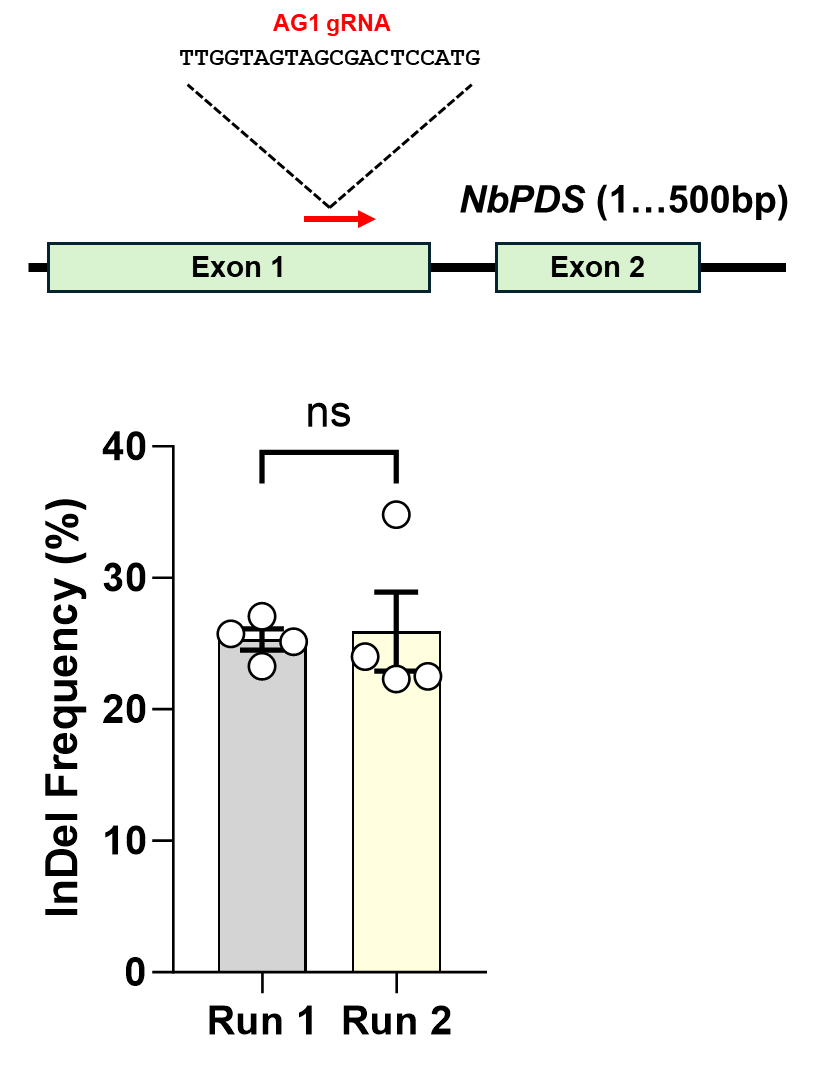


**Figure S2.** Genome editing through transient expression of the same gRNA (AG1) across two independent experimental runs. The top panel shows the AG1 gRNA sequence targeting the *PDS* gene. The bottom panel shows bars representing the mean InDel frequency ± SEM. Run 1 was conducted as part of dataset 1 and was also displayed in Figure S1. Run 2 was a second experiment conducted independently of both datasets. Each data point represents a biological replicate. A nonparametric, unpaired *t*-test was conducted to determine if the differences were statistically significant. ns, not significant.


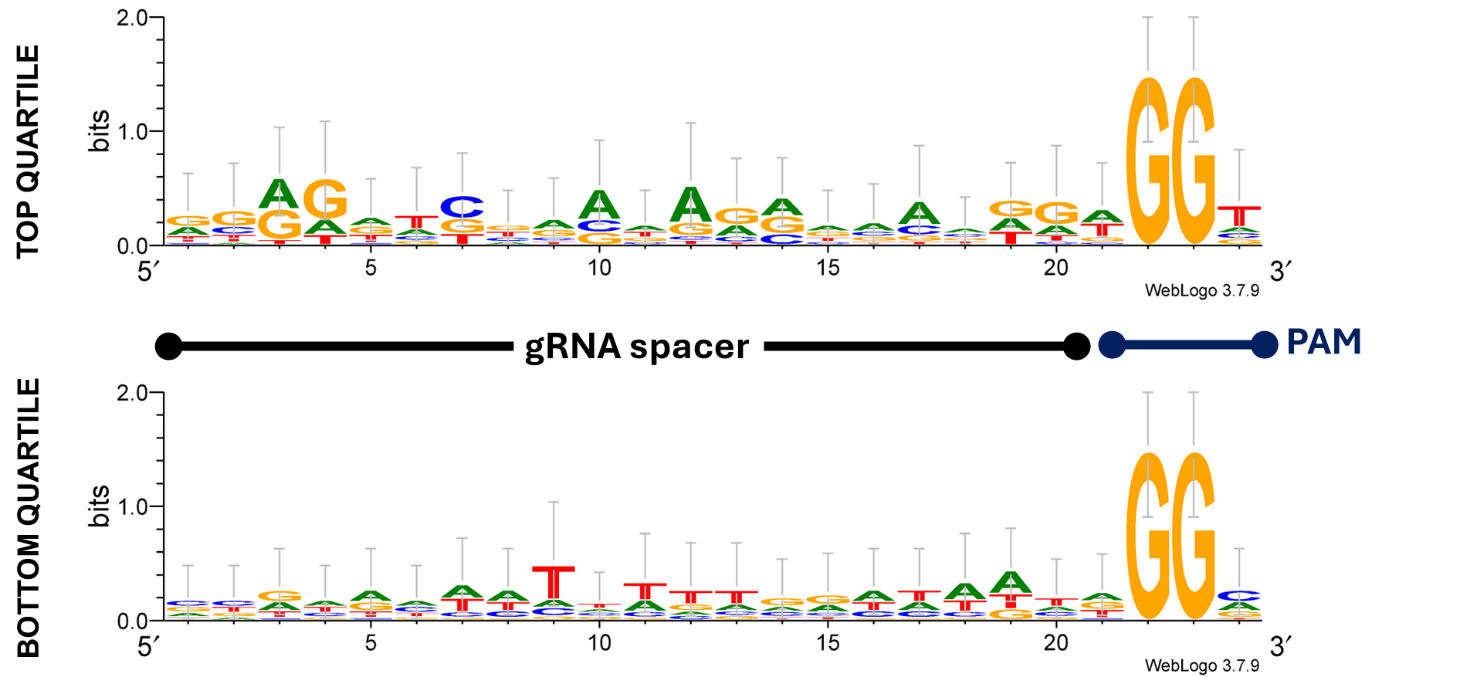


**Figure S3.** Analysis of gRNA sequence features to identify potential positional nucleotide preferences. The genome editing datasets in *N. benthamiana* was split into quartiles based on their AmpSeq-quantified genome editing efficiency. The gRNA spacer and the PAM sequence of gRNAs in the most and least efficient quartiles were combined from the two dataset and inputted into WebLogo3 to produce sequence logos. Bits represent the frequency of nucleotides at each position.


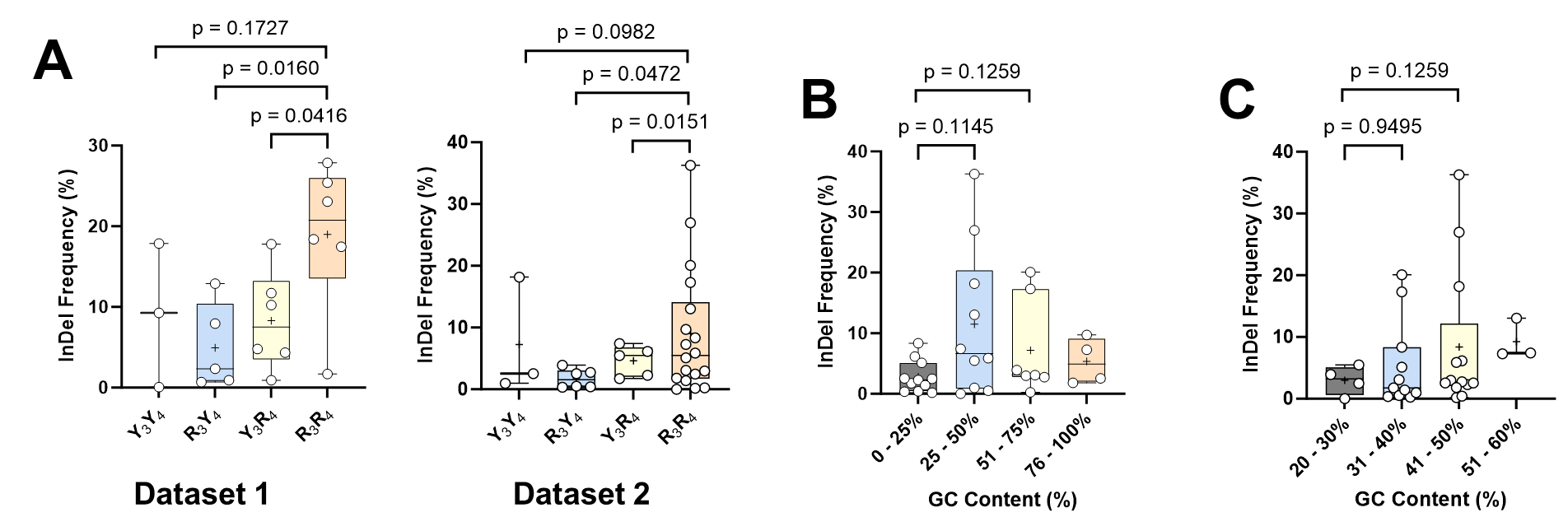


**Figure S4.** Testing the influence of positional nucleotide preference and GC content on gRNA activity. **(A)** Box and whisker plot of InDel frequencies for groups of gRNAs with pyrimidine nucleotides at both positions 3 and 4 (Y_3_Y_4_) of the 20 nt gRNA spacer, with purine nucleotides at both positions (R_3_R_4_) or with purines at position 3 and pyrimidine at position 4 (R_3_Y_4_) or with pyrimidines at position 3 and purines at position 4 in dataset 1 (left) and 2 (right). An unpaired *t*-test was conducted to test the differences between groups for dataset 1 and a Mann-Whitney U test was performed for dataset 2. **(B)** Box and whisker plot of InDel frequencies of gRNAs grouped by PAM distal GC content. **(C)** Box and whisker plot of InDel frequencies of gRNAs grouped by overall spacer GC content. The ‘+’ symbol represents the mean for all plots. A Mann-Whitney U test was performed to test whether differences between groups were of statistical significance for (B) and (C).


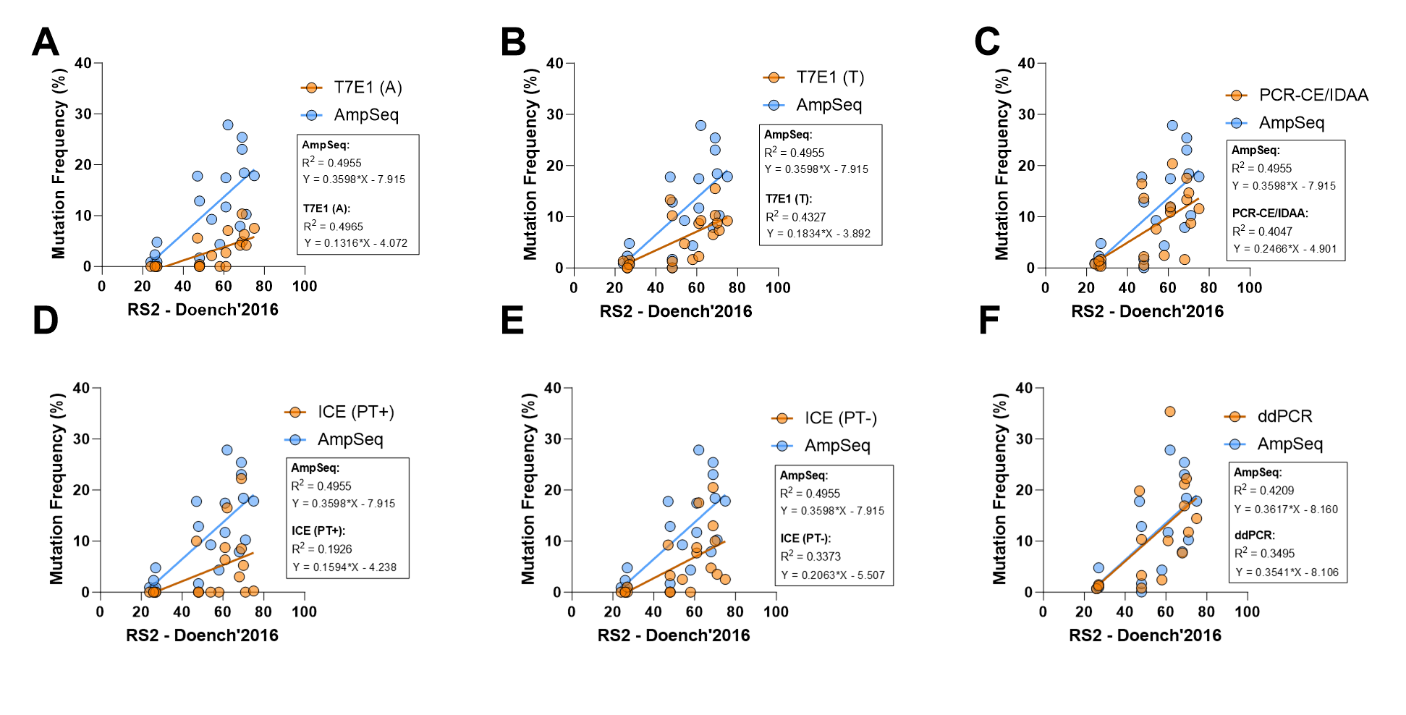


**Figure S5.** The use of different CRISPR genome editing quantification methods to generate the experimental dataset and its effect when evaluating gRNA on-target prediction scores as compared to AmpSeq. Linear regression analysis between genome editing efficiencies of gRNAs in dataset 1, quantified using **(A)** T7E1 (A), **(B)** T7E1 (T), **(C)** PCR-CE/IDAA, **(D)** Sanger sequencing-ICE (PT+), **(E)** Sanger sequencing-ICE (PT-), **(F)** ddPCR and the gRNA’s corresponding *Doench2016* on-target prediction scores were plotted on the same axis as when genome editing efficiencies were quantified using AmpSeq. The R^2^ and formula of the regression line are shown in the boxes.


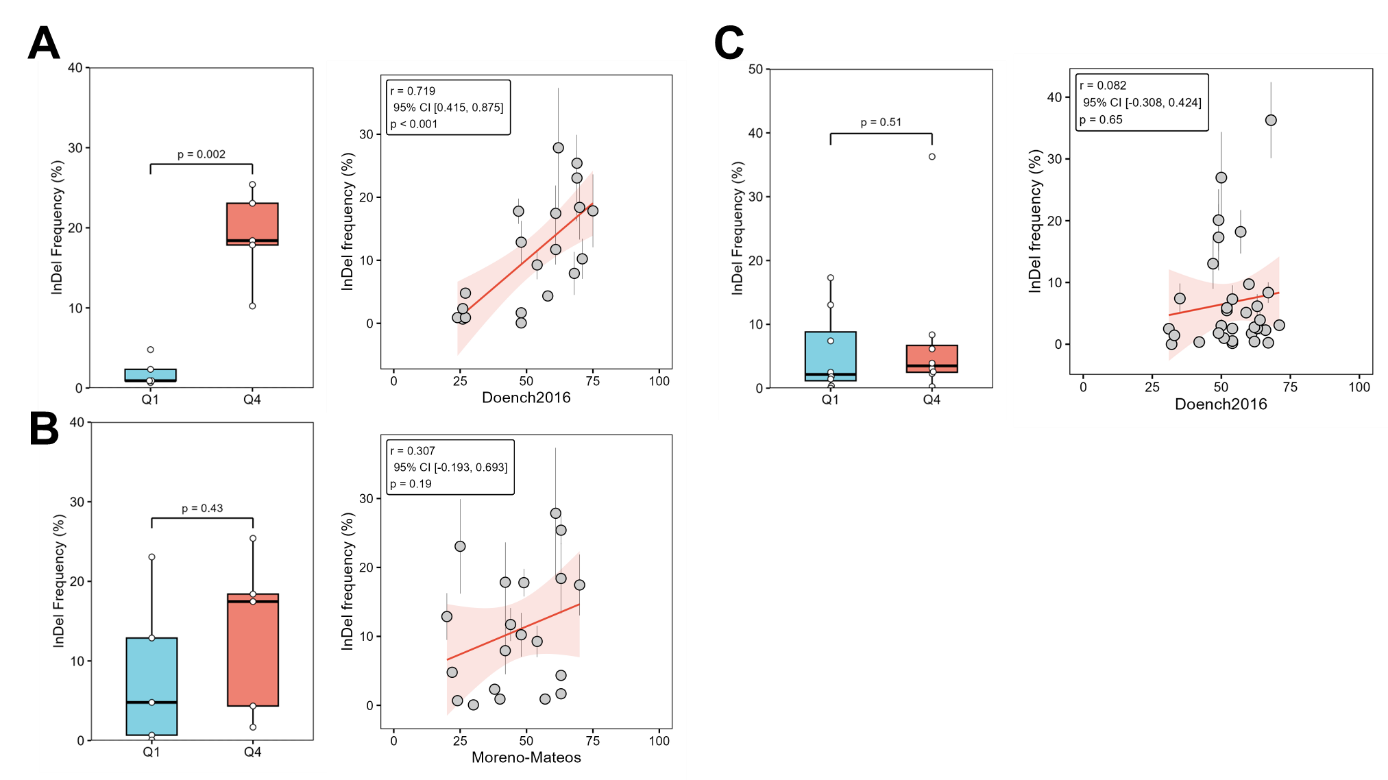


**Figure S6.** Evaluation of *Doench2016* and *Moreno-Mateos* efficiency scores and genome editing efficiency of gRNAs in dataset 1 and dataset 2. **(A)** Grouped (left) and correlation analysis (right) of *Doench2016* for gRNAs in dataset 1. gRNAs were split into quartiles and the InDel frequency for Q1 and Q4 were compared in the box and whisker plot (left). Linear regression and correlation analysis of *Doench2016* scores and experimental InDel frequencies for gRNAs in dataset 1 (right). Each data point represents the mean InDel frequency ± SEM of a gRNA and its corresponding prediction score. The shaded region represents the 95% CI for linear regression. The Spearman’s *r*, bootstrap 95% CI for the Spearman’s *r* and p-value was calculated and shown. **(B)** Grouped (left) and correlation analysis (right) of *Moreno-Mateos* for gRNAs in dataset 1. **(C)** Grouped (left) and correlation analysis (right) of *Doench2016* for gRNAs in dataset 2.


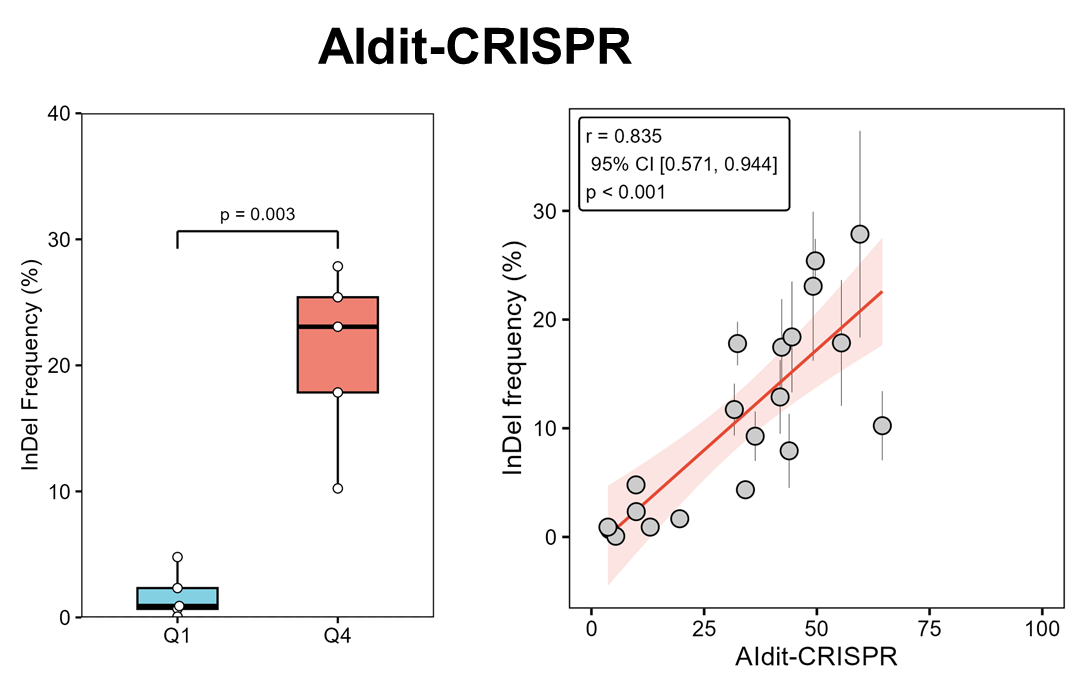


**Figure S7.** *AIdit-CRISPR* effectively predicts gRNA activity in dataset 1. Box and whisker plot (left) to compare the InDel frequencies of gRNAs grouped into the top quartile (Q4) by *AIdit-CRISPR* scores and ones grouped into the lowest quartile (Q1). An unpaired *t-*test was conducted to determine if differences were statistically significant. Linear regression and correlation analysis between *AIdit-CRISPR* efficiency prediction score and experimental gRNA activity in plants, measured as the InDel frequency. Each data point represents the mean InDel frequency ± SEM of a gRNA and its corresponding prediction score. The shaded region represents the 95% CI for linear regression. The Spearman’s *r*, bootstrapped 95% CI for the Spearman’s *r* and p-value was calculated and shown.


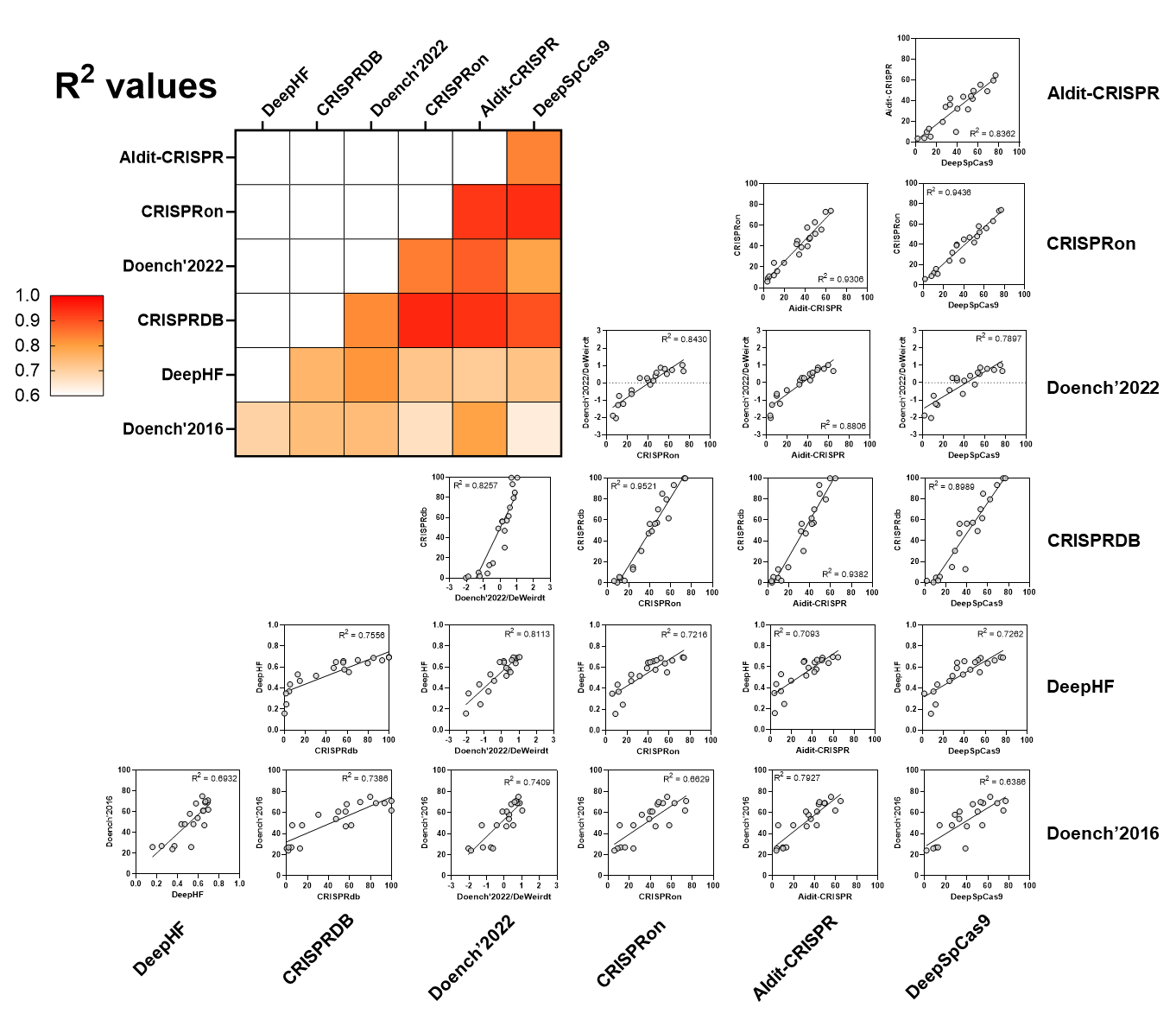


**Figure S8.** Linear regression analysis between the prediction scores of the 6 top-performing gRNA prediction scores for gRNAs in dataset 1. The *Doench2016* prediction score was also included for comparison. The R^2^ value for the linear regression analyses was represented using a heat map (top left)


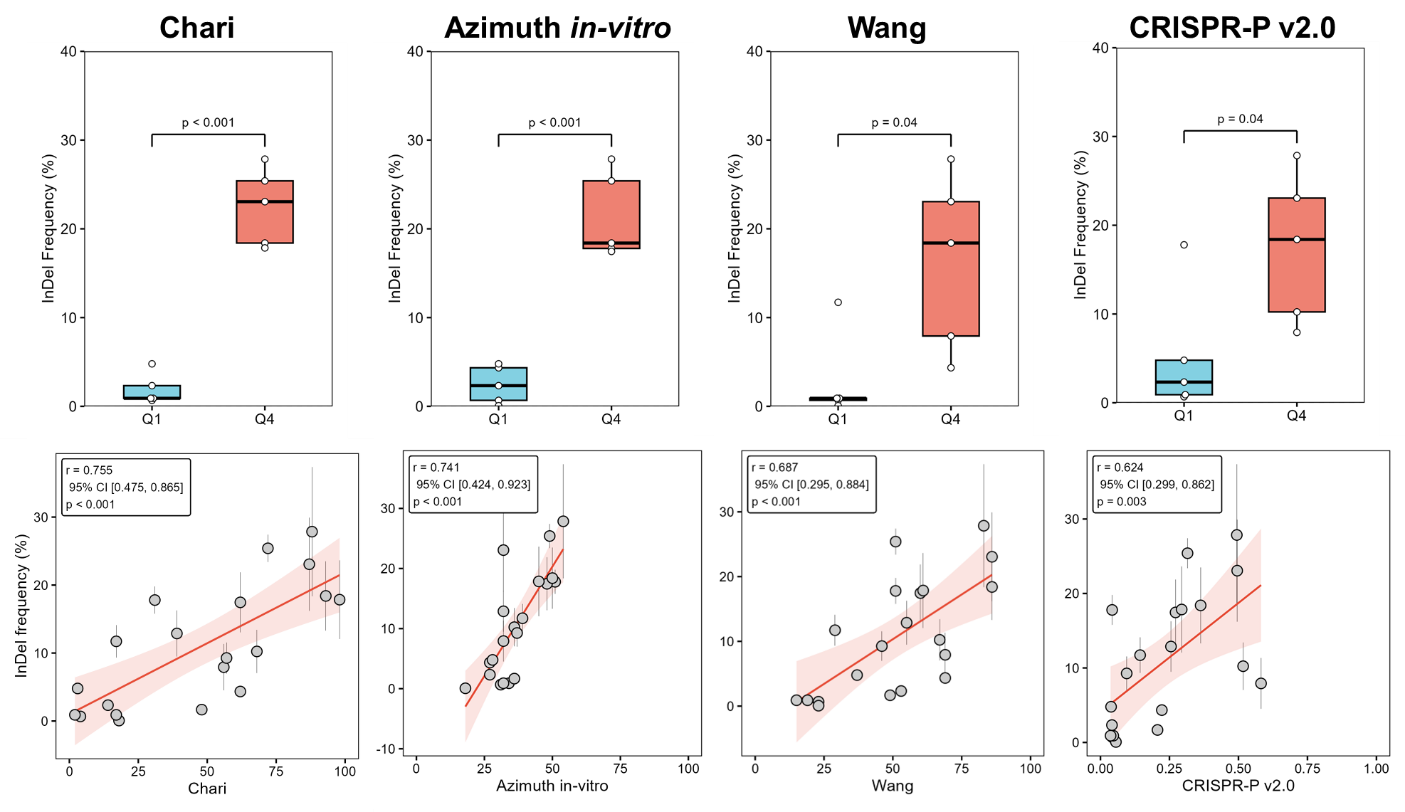


**Figure S9.** Four notable gRNA prediction tools on *CRISPOR* and *CRISPR-P v2.0*. Box and whisker plots (top panel) to compare the InDel frequencies of gRNAs grouped into the top (Q4) and bottom (Q1) quartiles by its prediction scores for dataset 1. An unpaired *t*-test was used to determine if differences between the InDel frequencies of the two quartiles are of statistical significance. Linear regression and correlation analysis (bottom panel) between the experimental InDel frequency and the prediction scores for gRNAs in dataset 1. Each data point represents the mean InDel frequency ± SEM of a gRNA and its corresponding prediction score. The regression line and its 95% CI are shown in red and shaded in light red, respectively. The Spearman’s *r,* bootstrapped 95% CI and p-value was calculated and shown.


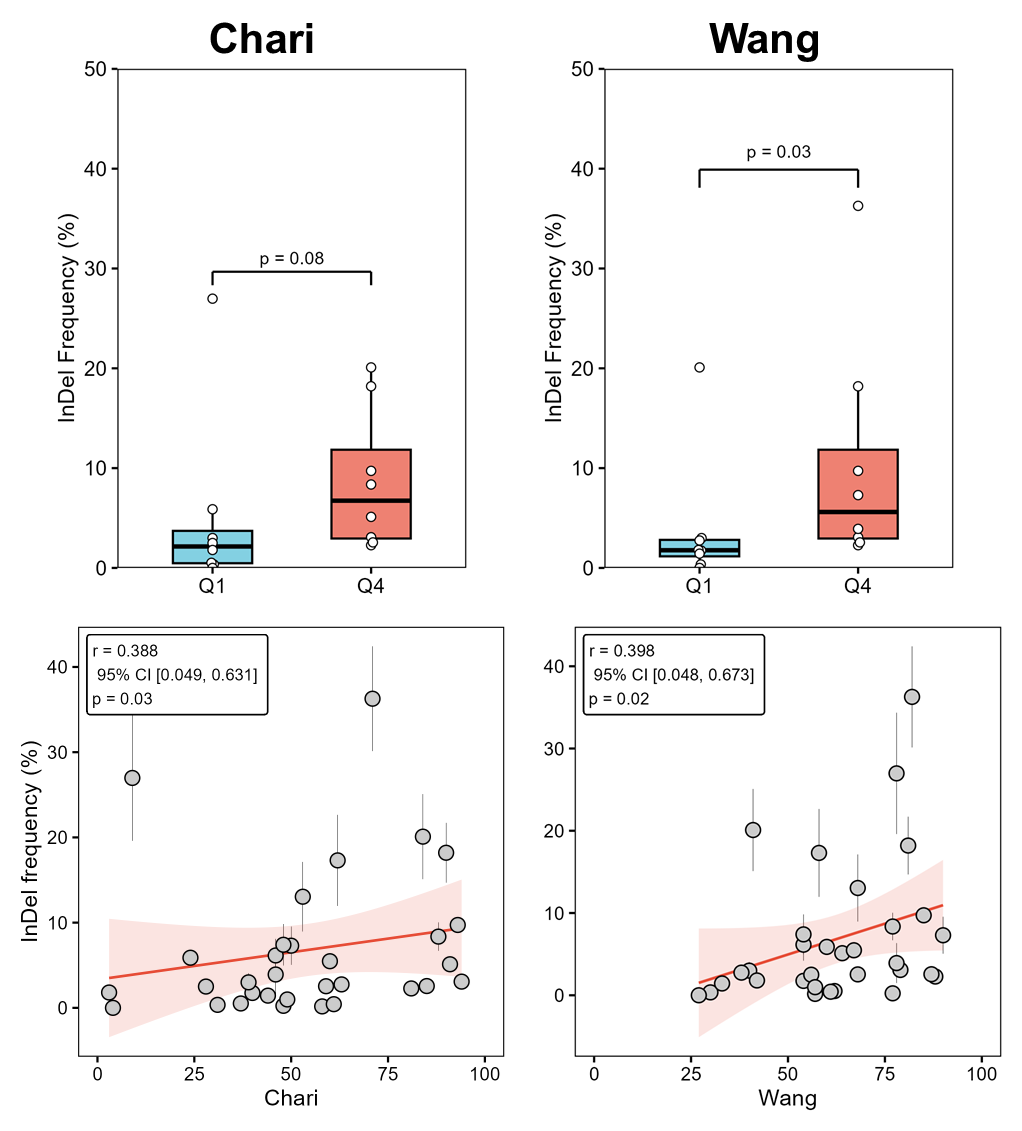


**Figure S10.** Evaluation of two notable gRNA prediction tools on *CRISPOR* for dataset 2. Box and whisker plots (top panel) to compare the InDel frequencies of gRNAs grouped into the top (Q4) and bottom (Q1) quartiles by its prediction scores. A Mann-Whitney U test was used to determine if differences between the InDel frequencies of the two quartiles are of statistical significance. Linear regression and correlation analysis (bottom panel) between the experimental InDel frequency and the prediction scores for gRNAs in dataset 2. Each data point represents the mean InDel frequency ± SEM of a gRNA and its corresponding prediction score. The regression line and its 95% CI are shown in red and shaded in light red, respectively. The Spearman’s *r,* bootstrapped 95% CI and p-value was calculated and shown.


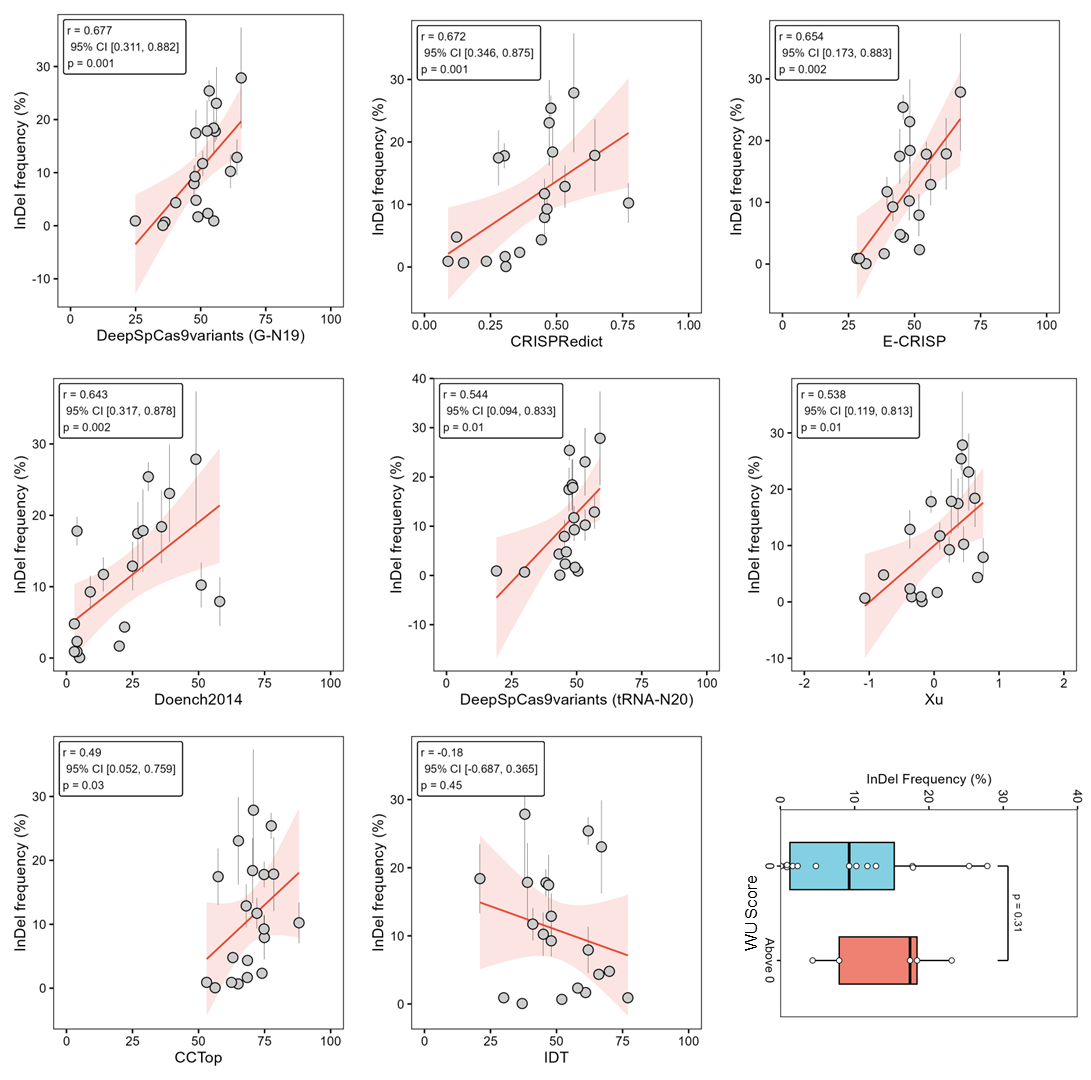


**Figure S11.** Evaluation of remaining gRNA on-target prediction tools using the GE dataset from dataset 1. Linear regression and correlation analysis between the gRNA prediction scores for each of the remaining 8 prediction tools and the gRNAs’ *in planta* genome editing efficiency, as quantified using AmpSeq. Each data point represents the mean InDel frequency ± SEM of a gRNA and its corresponding prediction score. The regression line and its 95% CI are shown in red and shaded in light red, respectively. The Spearman’s *r,* bootstrapped 95% CI and p-value was calculated and shown. For the *WU* prediction tool, gRNAs were grouped based on having scores of 0 (N = 15) or scores greater than 0 (N = 5). An unpaired *t*-test was conducted to test for the statistical significance of differences in the mean between the two groups.


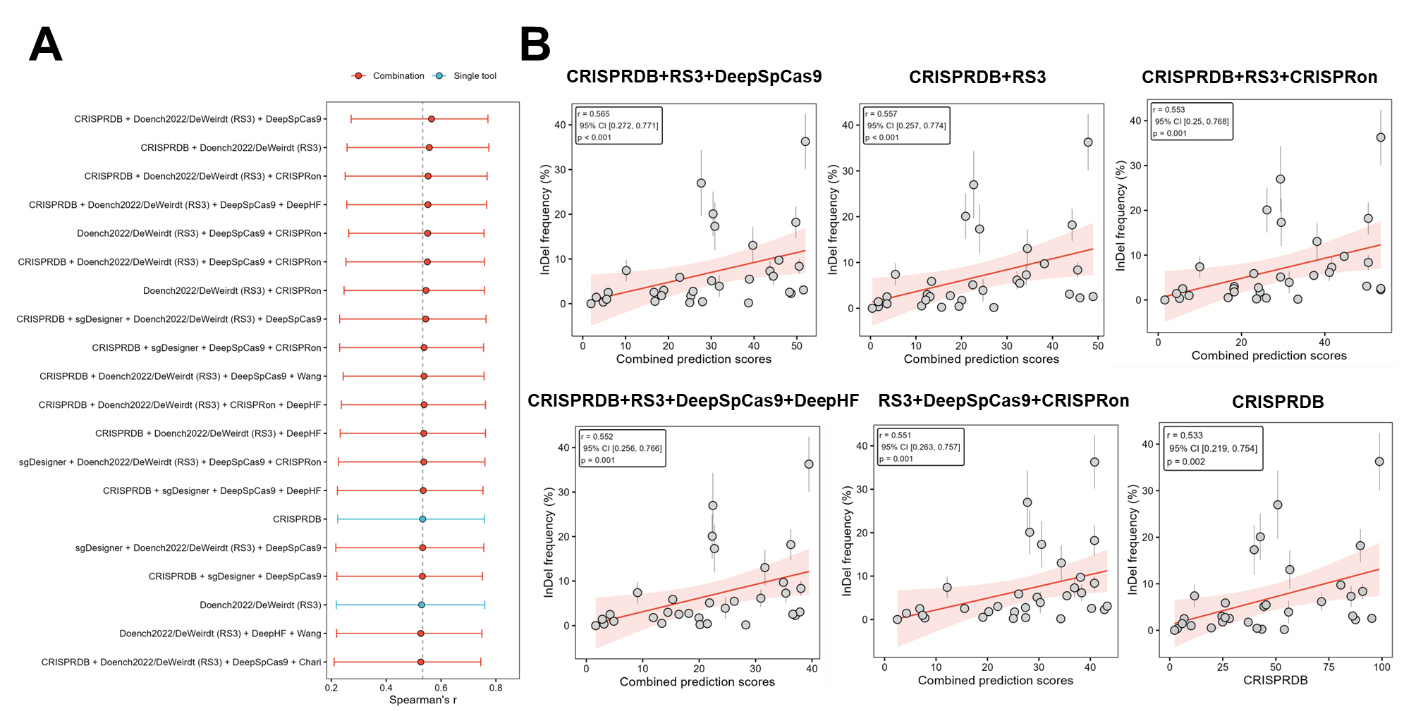


**Figure S12.** Testing ensembled efficiency scores for improved prediction. **(A)** Forest plot showing the top 20 ensembled combinations and individual tools with the highest Spearman’s *r*. The dotted line represents the Spearman’s *r* of *CRISPRDB*. Ensembled combinations of tools are coloured in red while individual tools are coloured in blue. **(B)** Linear regression and correlation analysis between ensembled prediction scores combined from multiple tools with experimental gRNA activity in dataset 2. The top five combinations with highest Spearman’s *r* are shown. Each data point represents the mean InDel frequency ± SEM of a gRNA and its corresponding prediction score. The regression line and its 95% CI are shown in red and shaded in light red, respectively. The Spearman’s *r*, bootstrapped 95% CI and p-value was calculated and shown.

**SUPPLEMENTARY TABLES**

**Table S1.** Table summarizing information for the gRNA prediction tools tested in this study.

| Prediction tool | Version on access | Access dates | Access portal | Website URL | Additional Info | Reference |
| --- | --- | --- | --- | --- | --- | --- |
| *Doench2014* | Dataset 1: CRISPOR v5.10  Dataset 2: CRISPOR v5.20 | Dataset 1: April 2024 | CRISPOR | http://crispor.gi.ucsc.edu/crispor.py |  | (Doench et al. 2014) |
| *Doench2016* | Dataset 1: CRISPOR v5.10  Dataset 2: CRISPOR v5.20 | Dataset 1: April 2024  Dataset 2: December 2025 | CRISPOR | http://crispor.gi.ucsc.edu/crispor.py |  | (Doench et al. 2016) |
| *Azimuth* in-vitro  (no longer accessible) | Dataset 1: CRISPOR v5.10 | Dataset 1: April 2024 | CRISPOR | http://crispor.gi.ucsc.edu/crispor.py |  | CRISPOR |
| *Doench2022/DeWeirdt* (*RS3*) | Dataset 1: CRISPick v2023-09-22  Dataset 2: CRISPick v2025-12-12 | Dataset 1: April 2024  Dataset 2: December 2025 | CRISPick | https://portals.broadinstitute.org/gppx/crispick/public | CRISPRko mode with Hsu (2013) tracrRNA selected. | (DeWeirdt et al. 2022) |
| *Chari* | Dataset 1: CRISPOR v5.10  Dataset 2: CRISPOR v5.20 | Dataset 1: April 2024  Dataset 2: December 2025 | CRISPOR | http://crispor.gi.ucsc.edu/crispor.py |  | (Chari et al. 2015) |
| *Xu* | Dataset 1: CRISPOR v5.10  Dataset 2: CRISPOR v5.20 | Dataset 1: April 2024 | CRISPOR | http://crispor.gi.ucsc.edu/crispor.py |  | (Xu et al. 2015) |
| *Wang* | Dataset 1: CRISPOR v5.10  Dataset 2: CRISPOR v5.20 | Dataset 1: April 2024  Dataset 2: December 2025 | CRISPOR | http://crispor.gi.ucsc.edu/crispor.py |  | (Wang et al. 2014) |
| *Moreno-Mateos* | Dataset 1: CRISPOR v5.10  Dataset 2: CRISPOR v5.20 | Dataset 1: April 2024 | CRISPOR | http://crispor.gi.ucsc.edu/crispor.py |  | (Moreno-Mateos et al. 2015) |
| *CCTop* | Dataset 1: CRISPOR v5.10  Dataset 2: CRISPOR v5.20 | Dataset 1: April 2024 | CRISPOR | http://crispor.gi.ucsc.edu/crispor.py |  | (Stemmer et al. 2015) |
| *WU* | Dataset 1: CRISPOR v5.10  Dataset 2: CRISPOR v5.20 | Dataset 1: April 2024 | CRISPOR | http://crispor.gi.ucsc.edu/crispor.py |  | (Wong et al. 2015) |
| *CRISPR-P v2.0* | Dataset 1: CRISPR-P v2.0 | Dataset 1: April 2024 | CRISPR-P server | http://crispr.hzau.edu.cn/CRISPR2/ |  | (Lei et al. 2014; Liu et al. 2017) |
| *CRISPRon* | Dataset 1 & 2: CRISPRon v1.0 | Dataset 1: April 2024  Dataset 2: December 2025 | CRISPRon server | https://rth.dk/resources/crispr/crispron/ |  | (Xiang et al. 2021) |
| *IDT on-target* | Dataset 1: No version information | Dataset 1: April 2024 | IDT server | https://sg.idtdna.com/site/order/designtool/index/CRISPR_CUSTOM |  | IDT |
| *E-CRISP* | Dataset 1: E-CRISP v5.4 | Dataset 1: April 2024 | E-CRISP server | http://www.e-crisp.org/E-CRISP/ |  | (Heigwer et al. 2014) |
| *DeepSpCas9* | Dataset 1 & 2: No version information | Dataset 1: April 2024  Dataset 2: December 2025 | DeepGE  server | https://deepcrispr.info/DeepSpCas9/ |  | (Kim et al. 2019) |
| *DeepSpCas9variants* | Dataset 1: No version information | Dataset 1: April 2024 | DeepGE  server | https://deepcrispr.info/DeepSpCas9variants/ |  | (Kim et al. 2020) |
| *DeepHF* | Dataset 1 & 2: No version information | Dataset 1: April 2024  Dataset 2: December 2025 | DeepHF | http://www.deephf.com/#/home | SpCas9_U6 mode. | (Wang et al. 2019) |
| *sgDesigner* | Dataset 1 & 2: No version information | Dataset 1: April 2024  Dataset 2: December 2025 | CRISPRDB | https://crisprdb.org/wu-crispr-website/ |  | (Hiranniramol et al. 2020) |
| *CRISPRDB* | Dataset 1 & 2: No version information | Dataset 1: April 2024  Dataset 2: December 2025 | CRISPRDB | https://crisprdb.org/custom.html | Custom prediction with U6 promoter. | (Chen and Wang 2022) |
| *CRISPRedict* | Dataset 1: No version information | Dataset 1: April 2024 | CRISPRedict | http://www.crispredict.org/ | U6 mode. | (Konstantakos et al. 2022) |
| *AIdit-CRISPR* | Dataset 1: No version information | Dataset 1: April 2024 | AIdit | https://crispr-aidit.com/webServer/gRNA |  | (Zhang et al. 2023) |

**REFERENCES**

CHARI, R., MALI, P., MOOSBURNER, M. & CHURCH, G. M. 2015. Unraveling CRISPR-Cas9 genome engineering parameters via a library-on-library approach. *Nature Methods,* 12**,** 823-826.

CHARI, R., YEO, N. C., CHAVEZ, A. & CHURCH, G. M. 2017. sgRNA Scorer 2.0: A Species-Independent Model To Predict CRISPR/Cas9 Activity. *ACS Synthetic Biology,* 6**,** 902-904.

CHEN, Y. & WANG, X. 2022. Evaluation of efficiency prediction algorithms and development of ensemble model for CRISPR/Cas9 gRNA selection. *Bioinformatics,* 38**,** 5175-5181.

CLEMENT, K., REES, H., CANVER, M. C., GEHRKE, J. M., FAROUNI, R., HSU, J. Y., COLE, M. A., LIU, D. R., JOUNG, J. K., BAUER, D. E. & PINELLO, L. 2019. CRISPResso2 provides accurate and rapid genome editing sequence analysis. *Nature Biotechnology,* 37**,** 224-226.

CONCORDET, J.-P. & HAEUSSLER, M. 2018. CRISPOR: intuitive guide selection for CRISPR/Cas9 genome editing experiments and screens. *Nucleic Acids Research,* 46**,** W242-W245.

CORSI, G. I., ANTHON, C. & GORODKIN, J. 2023. Letter to the editor: Testing on external independent datasets is necessary to corroborate machine learning model improvement. *Bioinformatics,* 39**,** btad327.

DEWEIRDT, P. C., MCGEE, A. V., ZHENG, F., NWOLAH, I., HEGDE, M. & DOENCH, J. G. 2022. Accounting for small variations in the tracrRNA sequence improves sgRNA activity predictions for CRISPR screening. *Nature Communications,* 13**,** 5255.

DOENCH, J. G., FUSI, N., SULLENDER, M., HEGDE, M., VAIMBERG, E. W., DONOVAN, K. F., SMITH, I., TOTHOVA, Z., WILEN, C. & ORCHARD, R. 2016. Optimized sgRNA design to maximize activity and minimize off-target effects of CRISPR-Cas9. *Nature biotechnology,* 34**,** 184-191.

DOENCH, J. G., HARTENIAN, E., GRAHAM, D. B., TOTHOVA, Z., HEGDE, M., SMITH, I., SULLENDER, M., EBERT, B. L., XAVIER, R. J. & ROOT, D. E. 2014. Rational design of highly active sgRNAs for CRISPR-Cas9–mediated gene inactivation. *Nature Biotechnology,* 32**,** 1262-1267.

GONG, Z., ZHANG, Y., XIA, D., YOON, S., CRISP, P. A. & BOTELLA, J. R. 2025. Comprehensive benchmarking of genome editing quantification methods for plant applications. *iScience,* 28.

HAEUSSLER, M., SCHÖNIG, K., ECKERT, H., ESCHSTRUTH, A., MIANNÉ, J., RENAUD, J.-B., SCHNEIDER-MAUNOURY, S., SHKUMATAVA, A., TEBOUL, L. & KENT, J. 2016. Evaluation of off-target and on-target scoring algorithms and integration into the guide RNA selection tool CRISPOR. *Genome biology,* 17**,** 1-12.

KONSTANTAKOS, V., NENTIDIS, A., KRITHARA, A. & PALIOURAS, G. 2022. CRISPR–Cas9 gRNA efficiency prediction: an overview of predictive tools and the role of deep learning. *Nucleic Acids Research,* 50**,** 3616-3637.

WANG, T., WEI, J. J., SABATINI, D. M. & LANDER, E. S. 2014. Genetic screens in human cells using the CRISPR-Cas9 system. *Science,* 343**,** 80-4.

XIANG, X., CORSI, G. I., ANTHON, C., QU, K., PAN, X., LIANG, X., HAN, P., DONG, Z., LIU, L. & ZHONG, J. 2021. Enhancing CRISPR-Cas9 gRNA efficiency prediction by data integration and deep learning. *Nature communications,* 12**,** 3238.

XU, H., XIAO, T., CHEN, C.-H., LI, W., MEYER, C. A., WU, Q., WU, D., CONG, L., ZHANG, F., LIU, J. S., BROWN, M. & LIU, X. S. 2015. Sequence determinants of improved CRISPR sgRNA design. *Genome Research,* 25**,** 1147-1157.
